# Ectopic Expression of *Glycine max* *GmNAC109* Enhances Drought Tolerance and ABA Sensitivity in *Arabidopsis*

**DOI:** 10.3390/biom9110714

**Published:** 2019-11-07

**Authors:** Nguyen Cao Nguyen, Xuan Lan Thi Hoang, Quang Thien Nguyen, Ngo Xuan Binh, Yasuko Watanabe, Nguyen Phuong Thao, Lam-Son Phan Tran

**Affiliations:** 1 School of Biotechnology, International University—Vietnam National University HCMC, Ho Chi Minh 700000, Vietnam; nguyencaonguyenbiotech@gmail.com (N.C.N.); htlxuan@hcmiu.edu.vn (X.L.T.H.); quang.nguyen212@gmail.com (Q.T.N.); 2 Faculty of Biotechnology and Food Technology, Thai Nguyen University of Agriculture and Forestry, Thai Nguyen 250000, Vietnam; ngobinh2014@gmail.com; 3 Stress Adaptation Research Unit, RIKEN Center for Sustainable Resource Science, 1-7-22, Suehiro-cho, Tsurumi, Yokohama 230-0045, Japan; yasuko.watanabe@riken.jp; 4 Institute of Research and Development, Duy Tan University, 03 Quang Trung, Da Nang, Vietnam; Stress Adaptation Research Unit, RIKEN Center for Sustainable Resource Science, 1-7-22, Suehiro-cho, Tsurumi, Yokohama 230-0045, Japan

**Keywords:** ABA, *Arabidopsis*, drought tolerance, ectopic expression, *Glycine max*, *GmNAC109*

## Abstract

The NAC (NAM, ATAF1/2, CUC2) transcription factors are widely known for their various functions in plant development and stress tolerance. Previous studies have demonstrated that genetic engineering can be applied to enhance drought tolerance via overexpression/ectopic expression of *NAC* genes. In the present study, the dehydration- and drought-inducible *GmNAC109* from *Glycine max* was ectopically expressed in *Arabidopsis* (*GmNAC109-*EX) plants to study its biological functions in mediating plant adaptation to water deficit conditions. Results revealed an improved drought tolerance in the transgenic plants, which displayed greater recovery rates by 20% to 54% than did the wild-type plants. In support of this finding, *GmNAC109*-EX plants exhibited lower water loss rates and decreased endogenous hydrogen peroxide production in leaf tissues under drought, as well as higher sensitivity to exogenous abscisic acid (ABA) treatment at germination and early seedling development stages. In addition, analyses of antioxidant enzymes indicated that *GmNAC109-*EX plants possessed stronger activities of superoxide dismutase and catalase under drought stress. These results together demonstrated that GmNAC109 acts as a positive transcriptional regulator in the ABA-signaling pathway, enabling plants to cope with adverse water deficit conditions.

## 1. Introduction

As the world’s population continues to grow rapidly, there has been a great demand for improved crop productivity to maintain global food security. Unfortunately, biotic and abiotic stresses have been putting significant pressure on crop growers [1,2]. Having been defined as adverse non-living environmental conditions, abiotic stresses—including drought, salinity, flooding and extreme temperatures—negatively influence the survival and productivity of crop plants [3]. Amongst the abiotic stressors, drought is believed to be the most threatening factor, posing great challenges to crop production and quality across the globe [4]. Drought, which can last for months, or years in the worst cases, may happen due to the shortages of rainfall or water in soil [5]. Prolonged water scarcity can lead to major physiological and developmental changes, causing impairment in plant development or even lethality [6]. For example, it has been shown that when plants are being exposed to drought, stomatal closure is induced which results in a reduction in photosynthetic activities [7]. Although drought-related phenotypic traits (e.g., root and shoot traits) of plants are often used to evaluate their resistance to drought, studies on molecular mechanisms employed by plants to react to drought have emerged as enormously important research topics [8,9,10,11]. It has been reported that the expression of many genes, including both functional and regulatory ones, is greatly altered when plants are exposed to water deficit conditions [12]. Gaining knowledge on the functions of drought-responsive genes would provide critical information for developing novel cultivars with improved drought tolerance by genetic engineering [13,14,15].

Being able to regulate gene expression, transcription factors (TFs) have been known to be indispensable players in the regulation of plant drought responses [16,17]. Among the drought-related TF families in plants, the NAC (NAM—No Apical Meristem of petunia, ATAF1/2—*Arabidopsis* Transcription-Activating Factor1/2, and CUC2—Cup-Shaped Cotyledon2) family has been identified as a key plant-specific TF family [18,19,20,21]. The NAC proteins are characterized by the possession of a conserved DNA-binding domain at the N-terminus and a transcriptional regulatory domain at the C-terminus [22]. In addition, a considerable number of *NAC* genes from various plant species have been evidenced to play imperative roles in mediating plant adaptation to drought [12,21]. For instance, transgenic *Arabidopsis* plants ectopically expressing *Cicer arietinum CarNAC2* [23] or *Suaeda liaotungensis SlNAC8* [24] display improved drought resistance. In rice (*Oryza sativa*), the drought tolerance can be enhanced by overexpressing the *OsNAC5* gene [25], or ectopically expressing the *Eleusine coracana EcNAC67* gene [26]. Applications of the *NAC* genes for improving drought resistance have been reported in many other crops as well, including tomato (*Solanum lycopersicum*) [27,28], banana (*Musa acuminata*) [29], cotton (*Gossypium hirsutum*) [30], and wheat *(Triticum aestivum*) [31,32].

In soybean (*Glycine max*), efforts have also been made to functionally characterize drought-related *GmNAC* genes. Amid more than 30 *GmNAC*s identified as drought- and/or dehydration-inducible genes [14,15,33,34,35], only a few genes have been characterized using *in planta* studies for their regulatory roles in mediating plant tolerance to water stress. For example, investigations have been undertaken on *GmNAC003*, *GmNAC004* [20], *GmNAC085* [36,37], and *GmSNAC49* (*Glycine max* stress-inducible *NAC49*) [38] in plant responses to the conditions of adverse water deficit. By performing a transcription activation assay in yeast, GmNAC109 was shown to possess an activation domain, which can act as a gene expression activator [35]. In addition, *GmNAC109* was proposed as a candidate for improvement of crops, in terms of drought tolerance, by using genetic engineering as its expression level was found to have a positive correlation with the drought-tolerant phenotype of soybean [14]. Previous studies also revealed that *GmNAC109* expression was up-regulated in the root tissues of soybean under both dehydration [33,35] and drought conditions [14]. Therefore, in the present study, we studied the drought-mediated actions of GmNAC109 in conferring drought resistance by analyzing the performance of *Arabidopsis* plants ectopically expressing *GmNAC109* (*GmNAC109-*EX) under drought and abscisic acid (ABA) treatments in terms of phenotypic, physiological, biochemical, and molecular aspects.

## 2. Materials and Methods

### 2.1. Generation of Transgenic Arabidopsis GmNAC109-EX Plants

The complete open reading frame of *GmNAC109* (*Glyma14g24220.1* in [33], or the so-called *GmNAC012* in [35]) was inserted downstream of the constitutive *CaMV 35S* promoter in the vector pGHX that has the pGreen plasmid backbone [39]. All the cloning procedures were performed as previously described [37,40], and a hygromycin selection marker was employed for the selection of transgenic plants instead of a kanamycin one. The recombinant vector was transferred into *Agrobacterium* followed by transformation of *Arabidopsis* (Col-0) using the floral dip method [41]. Screening of independent homozygous progenies of *GmNAC109-*EX lines was achieved by following the Mendelian genetic laws, which are based on the ratios of hygromycin-resistant and -sensitive phenotypes over three successive T_1_ to T_3_ generations [42]. Expression of the transgene *GmNAC109* was examined by RT-qPCR using the *GmNAC109*-specific primers and *Actin2* as the house-keeping gene (Supplementary Table S1).

### 2.2. Plant Growth Conditions

The seeds were surface-sterilized and germinated on Murashige and Skoog (MS) plates (1% sucrose, 0.8% agar) [43]. In brief, the collected seeds were sterilized by ethanol (70% *v*/*v*) for 1 min and sodium hypochlorite (2% *v*/*v*) for 15 min, followed by rinsing with sterile distilled water to remove the chemical residues. The decontaminated seeds were sowed on MS plates, which were then kept under dark conditions for 48 h at 4 °C for stratification, and subsequently under normal growth conditions (22 °C, humidity 60%, 16-h-light/8-h-dark photoperiod, 70 μmol m^−2^ s^−1^) for 14 days until used for further purposes.

### 2.3. Growth Assessment of Transgenic Arabidopsis Plants under Normal Conditions

Fourteen-day-old seedlings were transplanted from MS plates to soil (Tribat, Saigon Xanh Biotechnology Ltd. Company, Ho Chi Minh City, Vietnam) and irrigated under normal growth conditions (described in Section 2.2) for 14 days. For evaluation of root length, the plants were taken out of soil for measuring the root length. The maximum rosette radius was measured based on the length of the longest leaf of individual plant [37], using Image-J software (https://imagej.nih.gov/ij/). Measuring the whole area of *Arabidopsis* rosettes was performed following the procedure described in a previous study [44] using PhotoshopCC 2019 (Adobe, San José, CA, USA). The experiments were conducted in three biological replicates for each genotype, with 5 plants per replicate.

### 2.4. Drought Tolerance Assay

Fourteen-day-old seedlings were transplanted from MS plates to soil. Subsequently, the plants were grown under the growth conditions described in Section 2.2 with normal irrigation for 14 days, before the watering was suspended for 12 days when the relative soil moisture content dropped to approximately 18% [45]. After the drought treatment, irrigation was re-applied for 3 days, and the survival rate of each genotype was then evaluated [46]. Relative soil water content was monitored over the studied drought period by using a Moisture Meter (TK-100G, Yieryi, Guangdong, China). The experiments were conducted in three biological replicates for each genotype, with 10 plants per replicate.

### 2.5. Water Loss Rate Determination

For determination of water loss rate (WLR), leaves were removed from the 28-day-old plants that were grown on MS plates for 14 days and then soil for an additional period of 14 days, as described in Section 2.3. The fresh weight (FW) of individual leaves was immediately recorded using an analytical balance (Sartorius, no. 68285437, Göttingen, Germany). These leaves were then left to air-dry on a laboratory bench for a period of 5 h. While being air-dried, the leaves were weighed every 30 min to determine the water loss rates over the treated time period using FW reduction [47]. The experiment was conducted using nine replicates for each genotype, with two largest leaves per plant for each replicate.

### 2.6. Assays for Endogenous Hydrogen Peroxide and ROS-Scavenging Enzyme Activities

The plant materials for these assays were collected on day 12 of the drought treatment (Section 2.4). All experiments were conducted in three biological replicates for each genotype. To measure the hydrogen peroxide (H_2_O_2_) content, H_2_O_2_ was extracted from the harvested leaves using the previously described method with minor modifications [48]. Briefly, 0.2 g of the leaf tissues, which was used as one replicate sample, was homogenized in the phosphate-buffered saline (PBS) solution (1.8 mL, 0.1 M, pH 7.4). After centrifugation (10,000 rpm, 10 min, 4 °C) of the tissue homogenate, a mixture containing the supernatant (1 mL) and reaction solution (1 mL of 0.1% titanium(III) sulfate and 20% sulfuric acid) was prepared for the spectrophotometric measurement at 410 nm. A standard curve was established to determine the H_2_O_2_ concentrations in the samples.

To determine antioxidant enzyme activities, the leaf tissues (0.2 g tissue per sample) were homogenized in 2 mL cold potassium phosphate buffer (1 M, pH 7.8) containing EDTA 0.1 M and 2% polyvinylpyrrolidone (molecular weight of 8000). The supernatant obtained after centrifugation was used to quantify total soluble protein content using the Bradford method [49]. Bovine serum albumin (Sigma, Saint Louis, MO, USA) was used to establish the standard curve. Activities of enzymes superoxide dismutase and catalase were measured following the procedures described in previous studies [50,51].

### 2.7. Expression Analysis of Antioxidant Enzyme-Encoding Genes

Plants that were grown 14 days on MS plates and then 7 days in soil pots were used for the dehydration treatment. The whole plant samples were collected at various time points (0, 5, and 8 h) during the dehydration period for total RNA isolation. Methods for RNA extraction, first-strand cDNA synthesis, and RT-qPCR were previously described [14]. Information of primers used for RT-qPCR reactions is provided in Supplementary Table S1. Relative expression levels were determined following the 2^−ΔΔCt^ method [52], with *Actin2* being used as the house-keeping gene. The experiment was performed using three biological replicates per genotype for each time point of sample collection.

### 2.8. ABA Sensitivity Assay

Effects of exogenous ABA on seed germination and early seedling development were evaluated by sowing the seeds on MS medium (1% sucrose, 0.8% agar) supplemented with various ABA concentrations (0, 0.3, and 0.5 µM) and incubating the plates under normal growth conditions as specified in Section 2.2. The assays were perfomed following the method described in Huang et al. [53]. For germination assay, after 2 days of stratification, the plates were incubated under normal growth conditions for 3 days, and germination rates were then recorded based on the emergence of the seed radicles, as observed under a magnifying glass (10×). The green cotyledon rates were determined after 7 days of incubation under normal growth conditions based on the appearance of green cotyledons in each seedling. Each experiment was conducted in three replicates for each genotype, with 100 seeds per replicate.

### 2.9. Statistical Analysis

The data were analyzed by using the Student’s *t*-test (significant differences with *p*-value < 0.05 were indicated by asterisks) for comparison between WT and each EX line, or one-way ANOVA using SPSS software (significant differences with *p*-value < 0.05 were indicated by different letters according to Duncan’s multiple range test) for comparison among all genotypes.

## 3. Results

### 3.1. Reduced Growth Phenotype of GmNAC109-EX Lines under Normal Conditions

**Figure 1:**
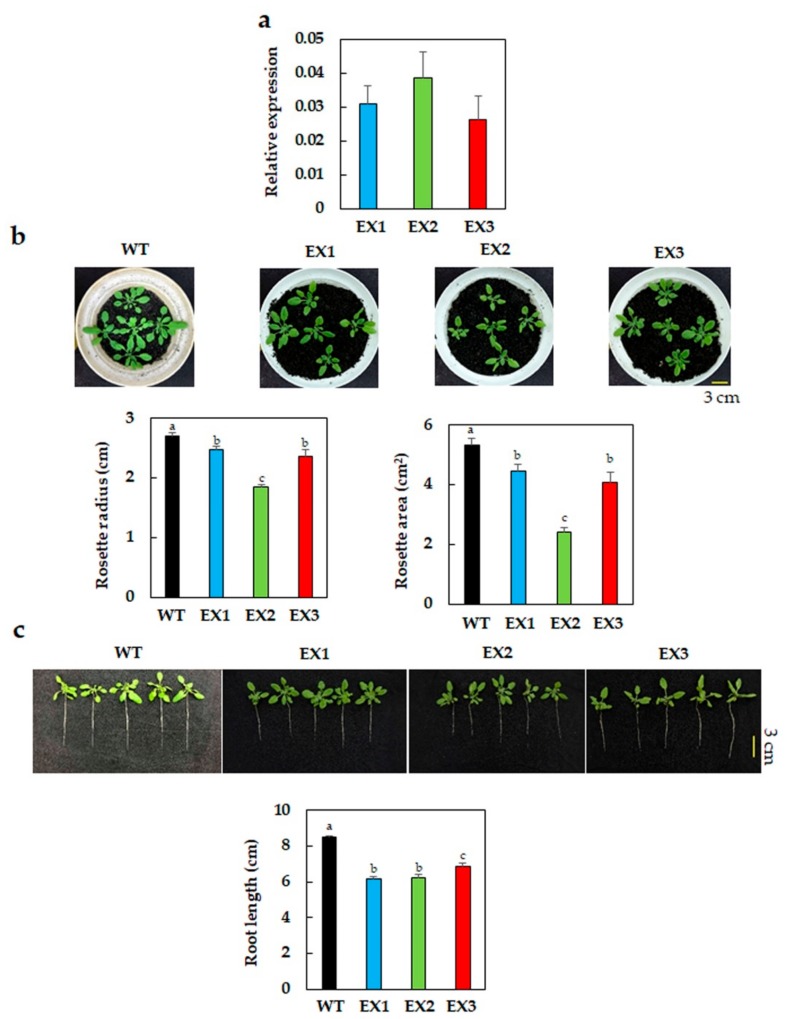
Transgene expression in the 28-day-old *GmNAC109*-EX1, 2, and 3 lines, and their phenotype under normal growth conditions. (**a**) Relative expression of the *GmNAC109* in the EX lines (*n* = 3 biological replicates/genotype). (**b**) Representative photographs, maximum rosette radius and average rosette area of 28-day-old EX lines (*n* = 3 replicates/genotype; 5 plants/replicate). (**c**) Representative photographs and average primary root length (*n* = 3 replicates/genotype; 5 plants/replicate). Standard errors were calculated and shown by error bars. Duncan’s multiple range test was used for statistical analysis, and significant differences among the genotypes were indicated by different letters (*p*-value < 0.05).

To explore the biological functions of GmNAC109, we introduced the complete coding sequence of this gene into the model plant *Arabidopsis* under the control of the constitutive promoter *CaMV 35S*. Three independent homozygous *GmNAC109*-EX lines (EX1, EX2, and EX3) that were successfully identified were firstly examined for the transgene expression and phenotypic characteristics (Figure 1). As expected, RT-qPCR using the *GmNAC109*-specific primers detected the expression of *GmNAC109* in all three EX lines but not in the WT plants, and the gene expression levels were comparable among the EX lines (Figure 1a). In comparison with WT, all three EX lines displayed smaller shoot-related phenotypes under normal growth conditions. As shown in Figure 1b, rosette-related traits, including their radius and area, were significantly reduced in the *GmNAC109*-EX plants. Additionally, the average primary root lengths of 4-week-old transgenic plants were ca. 2-cm shorter than that of WT (Figure 1c).

### 3.2. Improved Tolerance of GmNAC109-EX Plants to Drought

**Figure 2:**
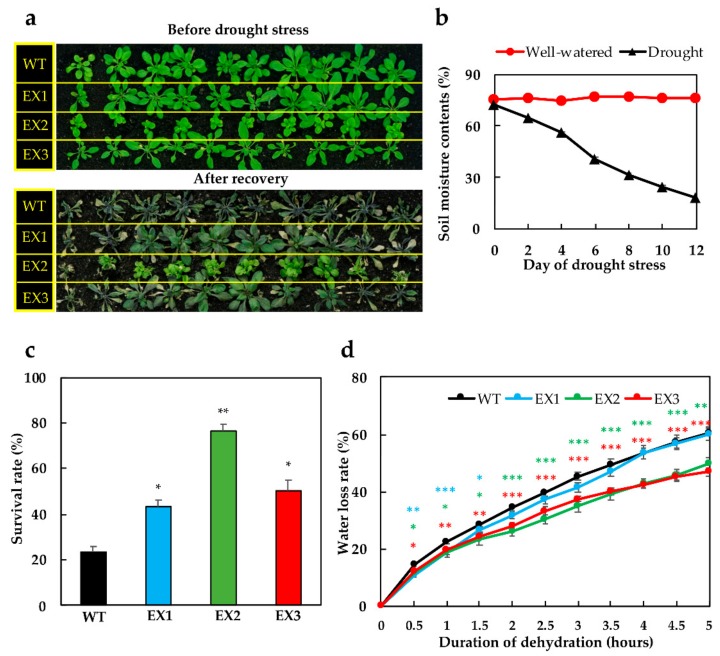
Examinations of drought survival rates and water loss rates of the *GmNAC109*-EX1, 2, and 3 lines. (**a**) Phenotypes of wild-type (WT) and EX lines before the drought treatment (i.e., 28-day-old) and after the recovery period (i.e., 43-day-old plants; 12-day-non-irrigation followed by 3-day re-watering). (**b**) Monitored relative soil moisture contents during the drought treatment are shown (*n* = 6 readings/time point). (**c**) Recorded survival rates of WT and EX plants after re-watering (*n* = 3 replicates/genotype; 10 plants/replicate). (**d**) Average water loss rates of leaves detached from 28-day old plants and left air-dehydrated over a 5-h duration (*n* = 9 replicates/genotype; 2 leaves/plant/replicate). Standard errors were calculated and shown by error bars. Asterisks indicate significant differences, as determined by a Student’s *t*-test, between each EX line and the WT (* *p*-value < 0.05; ** *p*-value < 0.01; *** *p*-value < 0.001).

The drought tolerance assay was conducted to assess the ultimate benefit brought by ectopic expression of *GmNAC109* in *Arabidopsis* plants. Results revealed that after 12 days of non-irrigation (i.e., when the relative soil moisture content dropped from 72% to ca. 18%) followed by 3-day re-watering, differential recovery rates were observed between *GmNAC109*-EX and WT plants (Figure 2a–c). More specifically, the *GmNAC109*-EX lines survived better than WT (43% of EX1, 77% of EX2, 50% of EX3, and only 23% of the WT plants) (Figure 2c). Next, the evaluation of the transpiration rates in individual leaf samples was undertaken using a dehydration-based method. We noted that the detached leaves dehydrated at bench showed a gradual reduction in their weight over the 5-h-dehydration (Figure 2d), indicating that they gradually lost water during the treatment. At the same time points of examination, the WT leaves suffered water loss at a higher rate than did the leaves of *GmNAC109*-EX plants. Specifically, during early dehydration treatment (0.5–1.5 h), excised WT leaves lost water faster than leaves of all three EX lines, while under prolonged dehydration conditions (2–5 h) they lost more water than the leaves of EX2 and EX3 lines (Figure 2d). These results imply that the *GmNAC109*-EX plants have better water retention under adverse water deficit conditions.

### 3.3. ABA Hypersensitivity of GmNAC109-EX Lines

**Figure 3:**
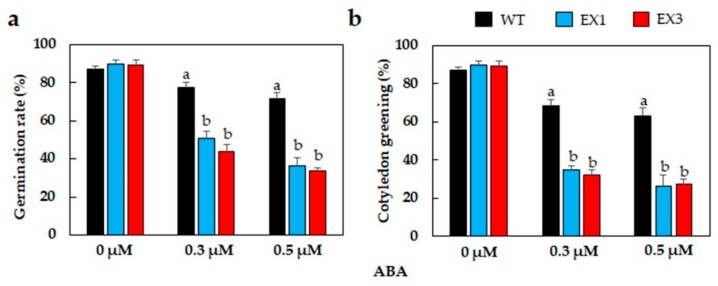
ABA sensitivity assay to examine ABA effects on seed germination and cotyledon greening of the *GmNAC109*-EX1 and 3 lines. (**a**) Germination rates of seeds on MS medium supplemented with 0, 0.3, and 0.5 μM ABA after 3 days of incubation under normal growth conditions (*n* = 3 replicates/genotype; 100 seeds/replicate). (**b**) Cotyledon greening rates at different ABA concentrations after 7 days of incubation under normal growth conditions (*n* = 3 replicates/genotype; 100 seeds/replicate). Standard errors were calculated and shown by error bars. Duncan’s multiple range test was used for statistical analysis of the data received from all genotypes within the same treatment, and significant differences among the genotypes were indicated by different letters (*p*-value < 0.05).

Phytohormones, particularly abscisic acid (ABA), have been known to participate in regulating plant acclimation to drought [54]. Therefore, we tested the effects of exogenous ABA application on the germination rate and seeding development (cotyledon greening) to find out if GmNAC109 belongs to the ABA-mediated signaling pathway in regulation of plant drought responses. As the EX2 line displayed a significantly smaller rosette in comparison with that of other two EX lines, we excluded this line from the ABA assay and subsequent experiments. Results of the germination assay revealed that on the ABA-free MS plates, WT and two examined EX lines shared similar germination rates (approximately 89%). However, an inhibitory effect of ABA on seed germination was observed as the proportions of seeds that could germinate were reduced markedly (Figure 3a). Comparing the germination rates between WT and the EX lines revealed that under the same ABA concentrations, the decline in germination rates was more pronounced in the EX plants. According to our data, WT seed germination dropped by 10% and 16% by 0.3 and 0.5 μM ABA application, respectively, whereas the corresponding values were 39% and 54% in EX1, and 45% and 55% in EX3. Consistently, the results of examination of the ABA effects on green cotyledon also showed stronger ABA-responsiveness of the *GmNAC109*-EX lines. As shown in Figure 3b, the proportion of WT seedlings that could display green cotyledons was more than double of that obtained from EX1 and EX3 seedlings at the same exogenous ABA concentrations.

### 3.4. Enhanced Antioxidant Enzyme Activities in the GmNAC109-EX Lines

**Figure 4:**
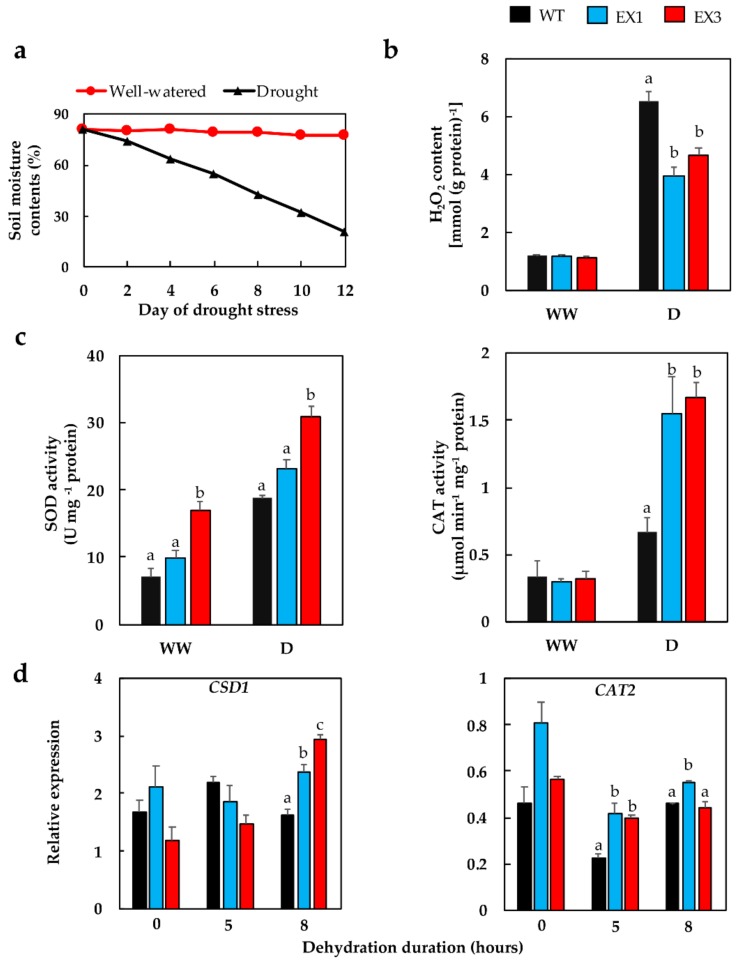
Drought-induced oxidative stress-related analyses in *GmNAC109*-EX1 and 3 lines. (**a**) Relative soil moisture contents were measured over the 12-day-drought duration (*n* = 6 readings/time point). (**b**) Hydrogen peroxide (H_2_O_2_) content in leaf tissues of 40-day-old plants that had been grown under a well-watered (WW) condition or exposed to a 12-day drought (D) (*n* = 3 biological replicates/genotype/treatment). (**c**) Antioxidant activities of superoxide dismutase (SOD) and catalase (CAT) enzymes in leaves of 40-day-old plants that had been grown under a well-watered (WW) condition or exposed to a 12-day drought (D) period (*n* = 3 biological replicates/genotype/treatment). (**d**) Relative expression of *CSD1* (*copper/zinc superoxide dismutase 1*) and *CAT2* (*catalase 2*) genes in 21-day-old plants that were subjected to 0-, 5-, and 8-h dehydration treatment (*n* = 3 biological replicates/genotype). Standard errors were calculated and shown by error bars. Duncan’s multiple range test was used for statistical analysis of the data received from all genotypes within the same treatment, and significant differences among the genotypes were indicated by different letters (*p*-value < 0.05).

Plant damage by oxidative stress is usually induced under extended drought conditions, which is featured by the accumulation of endogenous reactive oxygen species (ROS), such as hydrogen peroxide (H_2_O_2_) [55]. Therefore, we next investigated the accumulation levels of cellular H_2_O_2_ contents in the leaf tissues of plants growing under normal and drought conditions. Our data showed that under irrigation conditions, the healthy plants exhibited comparable H_2_O_2_ levels in the WT and EX lines (Figure 4a,b). The H_2_O_2_ concentrations showed increases in the stress-treated leaf samples by 5.4-fold, 3.3-fold, and 4.1-fold in WT, EX1, and EX3 lines, respectively (Figure 4a,b). This differential stress-induced H_2_O_2_ production resulted in a lower H_2_O_2_ excess in the EX plants, suggesting a less serious oxidative stress impact on EX plants in comparison with WT.

To cope with water deficit-induced oxidative damage, antioxidant defense is activated in plants to detoxify excessive ROS [36,37]. Therefore, we proceeded to analyze the activities of superoxide dismutase (SOD) and catalase (CAT), the two ROS-scavenging enzymes that are well known to be involved in plant acclimation to water stress [56,57], to further estimate the antioxidant defense capacity of *GmNAC109*-EX plants under drought. Under water deficit, the SOD activity in EX3 was approximately 1.6-fold higher than that in WT, whereas the CAT activities in both EX lines were increased by more than 2-fold compared with the corresponding activity found in the WT (Figure 4c). Additional examination of the expression patterns of *copper/zinc superoxide dismutase 1* (*CSD1*, encoding a SOD enzyme) and *Catalase 2* (*CAT2*, encoding a catalase enzyme) genes under dehydration conditions showed that *CSD1* expression was induced at a much higher level in the EX lines (by 1.5–1.8-fold) than in WT plants after 8-h dehydration (Figure 4d). Meanwhile, the differential expression levels in *CAT2* between the EX lines (1.8–1.9-fold higher) and WT were recognized at earlier stage of dehydration (5 h) (Figure 4d).

## 4. Discussion

*GmNAC109* has been highlighted as an important dehydration- and drought-inducible gene, as its expression has been found to be associated with drought tolerance in soybean [14,33,35]. Furthermore, comparative sequence analyses of soybean NACs with *Arabidopsis* and rice NACs depicted that GmNAC109 TF has a putative nuclear localization signal, while the yeast hybrid assay indicated this protein possesses transactivation capacity [35]. In this study, *Arabidopsis* plants ectopically expressing *GmNAC109* displayed remarkably improved tolerance to drought, with higher survival rates after a 12-day-drought imposition in comparison with the WT (Figure 2a–c). Enhancement of drought tolerance in transgenic plants by overexpressing/ectopically expressing *NAC* genes has been well documented in many previous studies, such as those that used tomato *SlNAC3* [58], rice *SNAC1* [59], *SNAC3* [8] and *ONAC022* [60], pumpkin (*Cucurbita moschata*) *CmNAC1* [61], and soybean *GmNAC085* [36,37] and *GmNACS49* [38]. Such transgenic studies have also revealed diverse mechanisms of the NAC TFs in contributing to the enhanced drought tolerance of the transgenic plants. The advantageously modified characteristics achieved by *NAC* overexpression/ectopic expression include faster ROS detoxification by enhanced enzymatic activities of SOD, CAT, ascorbate peroxidase, glutathione peroxidase, glutathione *S*-transferase and glyoxalases [36,37,62,63,64], more developed root systems [26,65], better cellular water reservation [37,66], and/or increased accumulation of proline and sugar contents [24,67].

It is worth noting that the ectopic expression of *GmNAC109* by using the *CaMV 35S* promoter led to the growth penalty in the *GmNAC109*-EX plants, especially smaller phenotypes of both shoot and root organs than what were observed in the WT (Figure 1). This observation is in accordance with the phenotypic appearance of *Arabidopsis* plants ectopically expressing *GmNAC085*, in which the transgene was also driven by the *35S* promoter [37]. In the present study, the *GmNAC109*-EX plants showed lower water loss rates than did the WT plants (Figure 2d), suggesting that the transgenic plants might evaporate less water from their smaller rosette (Figure 1b); and therefore, they were more tolerant to drought than WT plants. Such growth adjustment for better drought survival by saving limitedly available water amount was also observed in many transgenic plants, such as those overexpressing/ectopically overexpressing the *Arabidopsis cytokinin oxidase/dehydrogenase* (*CKX)* genes involved in cytokinin catabolism [68,69,70]. Importantly, the transgenic *Arabidopsis* plants carrying the construct *RD29A:GmNAC085*, in which the stress-inducible *RD29A* promoter was used to drive the ectopic expression of *GmNAC085*, displayed normal development, as well as a significant level of drought tolerance, when compared with that of WT plants [36]. Therefore, using an alternative promoter such as a stress-inducible promoter is likely to improve plant drought tolerance without compromising plant growth and development.

It has been known that NAC TFs regulate various plant developmental processes, as well as plant responses to both biotic and abiotic stress factors [71,72,73,74]. In regulating plant adaptation to drought, the NAC members participate in both ABA-dependent [75,76,77] and ABA-independent [78] pathways. From a previous study, GmNAC109 would appear to function in an ABA-independent manner as *GmNAC109* transcription was not altered upon exogenous ABA treatment [35]. In the current study, the findings obtained from the assays for ABA responsiveness revealed that the *GmNAC109*-EX lines showed ABA hypersensitivity on media supplied with various concentrations of ABA in an ABA dose-dependent manner (Figure 3). Analysis of the promoter region of *GmNAC109* conducted in our previously published study indicated that the *GmNAC109* promoter has multiple *cis*-acting elements, including an ABA-responsive element 2 (ABRE2, containing the core sequence “ACGTGG/TC”), a dehydration-responsive element (DRE, containing the core sequence “A/GCCGAC”) and two G-box elements (core sequence “CACGTG”) [14]. It has been known that ABA-responsive regulation may require a combination of ABRE and its partner [79]. *Cis*-regulatory elements that have been identified to couple with ABRE in facilitating ABA-mediated transcription include CE1 (coupling element 1), CE3, another ABRE element, or DRE [79,80,81]. In addition, DREs detected in the promoter regions of many drought-responsive genes have been found to function in ABA-independent regulatory system [78,82]. These data collectively imply that GmNAC109 activity might be governed by not only ABA-dependent but also ABA-independent signaling pathways in regulating plant responses to limited water conditions. In addition, the enhanced ABA responsiveness of the *GmNAC109*-EX plants (Figure 3) and their better ability to retain cellular water (Figure 2d) suggest the possible involvement of GmNAC109 in coordination with ABA to regulate the transpiration process in plants. ABA is the key phytohormone that controls the stomatal activities [79]. As drought progresses, endogenous ABA content is increased, even up to 30-fold [83,84], to facilitate the closure of stomatal aperture, thereby preventing water loss by transpiration [66]. Therefore, detailed functional analysis of GmNAC109 in stomatal movement is an interesting topic for future studies.

One of drought’s negative impacts is the over-production of endogenous ROS, as ROS at high levels are harmful to cellular structure and metabolism [55,85]. A study in wheat conducted under drought showed that around 70% of generated H_2_O_2_ came from photorespiration, a triggered activity due to the closure of stomata and intensive sunlight exposure [86]. Thus, H_2_O_2_ levels are usually measured in drought tolerance studies, and used as an indicative parameter for evaluating the oxidative damage in plants under limited water conditions [36]. In our study, lower H_2_O_2_ contents in drought-treated EX plants were observed (Figure 4b), which might be attributed to the higher CAT activities detected in the EX lines in comparison with WT plants under drought (Figure 4c). As SOD provide the first layer of antioxidant defense by dismutating superoxide radicals, the observation of enhanced SOD activities in the EX lines, when compared with that of WT plants, suggests the more pronounced protection of the EX lines from oxidative stress (Figure 4c) [85,87,88]. Furthermore, the higher degree of induction in *CSD1* and *CAT2* expression under dehydration conditions in the *GmNAC109*-EX lines compared with that of WT indicates a positive relationship between the enzyme activities and gene expression levels under water deficit conditions (Figure 4c,d). On the other hand, under well-watered conditions, the activity of SOD of EX3 line was higher than that of the WT, whereas the expression profile of *CSD1* was not significantly different between the two genotypes (Figure 4c,d). No direct correlation between CAT activities and *CAT2* expression was observed either under stress conditions, where the enzyme activities were enhanced yet *CAT2* expression was down-regulated in all genotypes (but still higher in EX versus WT plants) (Figure 4c,d). The reason could be that the expression of only one gene coding for an enzyme isoform was examined in our study, while there are more than one isoforms of these antioxidant enzymes, and the activity of a given enzyme is the sum of activities of its different isoforms [89]. Therefore, a comprehensive expression analysis of all members of the *SOD* and *CAT* gene families in the context of *GmNAC109* ectopic expression will be an interesting future study. In addition to GmNAC109, previous analyses also demonstrated that the *CSD1* and *CAT2* genes are under the regulation of other NAC proteins [37,90,91], inferring that NAC TFs are widely involved in modulation of antioxidant defense in response to stresses. Hence, ectopic expression of *GmNAC109* in *Arabidopsis* is able to alleviate the drought-induced oxidative damage in transgenic plants by boosting activities of ROS-scavenging enzymes, suggesting that GmNAC109 is involved in improvement of antioxidant defense in transgenic plants under water stress, as some other reported NAC TFs, such as SlNAC8 [24], SlNAC35 [27], GmNAC085 [37], and TaNAC29 [62].

## 5. Conclusions

Our study has elucidated the important role of GmNAC109 in mitigation of drought effects on plants. The improved tolerance of *GmNAC109*-EX plants was associated with reduced water loss rates and enhanced ROS-scavenging capability of SOD and CAT enzymes. Furthermore, our results provided evidence for the modulation of GmNAC109 action in an ABA-dependent manner.

## Supplementary Materials

The following is available online at https://www.mdpi.com/2218-273X/9/11/714/s1, Table S1: Genes and primers used for RT-qPCR analysis.
